# Establishing an implementation network: lessons learned from community-based participatory research

**DOI:** 10.1186/1748-5908-4-17

**Published:** 2009-03-31

**Authors:** Laurie A Lindamer, Barry Lebowitz, Richard L Hough, Piedad Garcia, Alfredo Aguirre, Maureen C Halpain, Colin Depp, Dilip V Jeste

**Affiliations:** 1 Department of Psychiatry, University of California, San Diego, La Jolla, California, USA; 2 Center of Excellence for Stress and Mental Health (CESAMH), VA San Diego Healthcare Systems, San Diego, California, USA; 3 Stein Institute for Research on Aging, University of California, La Jolla, California, USA; 4 Psychiatry and Family & Community Medicine, Center for Rural and Community Behavioral Health (CRCBH), University of New Mexico, School of Medicine, Dexter, New Mexico, USA; 5 San Diego County Adult and Older Adult Mental Health Services, San Diego, California, USA

## Abstract

**Background:**

Implementation of evidence-based mental health assessment and intervention in community public health practice is a high priority for multiple stakeholders. Academic-community partnerships can assist in the implementation of efficacious treatments in community settings; yet, little is known about the processes by which these collaborations are developed. In this paper, we discuss our application of community-based participatory research (CBPR) approach to implementation, and we present six lessons we have learned from the establishment of an academic-community partnership.

**Methods:**

With older adults with psychosis as a focus, we have developed a partnership between a university research center and a public mental health service system based on CBPR. The long-term goal of the partnership is to collaboratively establish an evidence-based implementation network that is sustainable within the public mental healthcare system.

**Results:**

In building a sustainable partnership, we found that the following lessons were instrumental: changing attitudes; sharing staff; expecting obstacles and formalizing solutions; monitoring and evaluating; adapting and adjusting; and taking advantage of emerging opportunities. Some of these lessons were previously known principles that were modified as the result of the CBPR process, while some lessons derived directly from the interactive process of forming the partnership.

**Conclusion:**

The process of forming of academic-public partnerships is challenging and time consuming, yet crucial for the development and implementation of state-of-the-art approaches to assessment and interventions to improve the functioning and quality of life for persons with serious mental illnesses. These partnerships provide necessary organizational support to facilitate the implementation of clinical research findings in community practice benefiting consumers, researchers, and providers.

## Background

Effective approaches to implementation of evidence-based practices in community settings necessarily involve close collaboration between the research team and the stakeholders, end-users, and policy-makers responsible for sustaining the new practices [1-3]. The need for such collaboration has been recognized by policymakers at the highest levels of executive decision-making, including, in the United States, the President's New Freedom Commission on Mental Health [4].

The nature of the collaboration between community partners and academic researchers varies based upon the relative distribution of power between the organizations [5-7]. Three approaches to power sharing have been described. One approach, "community-targeted" research, enlists the "voice" of the community to engage participants in studies that the researcher has selected and to aid in the dissemination of the research findings [5]. In a "community-based" research approach, the community participation is greater. The community has a "vote" in the selection of research topics, but the researcher often determines the study design, method of data collection, and analysis of data. In a "community-driven" research approach the decision-making for all aspects of the research is shared, giving the community partner equal power, and hence the ability to "veto".

Community-driven research methods are akin to community-based participatory research (CBPR), an approach that is solidly established in many areas of public health research. Its application to developing successful and sustainable mental health implementation networks, however, is minimal. In order to initiate an implementation network that bridges the gaps between an academic research center and a large public mental health system to create a collaborative implementation research network, we used the principles of community-based participatory research (CBPR) [8]. The overall purpose of the network is to enhance care for older people with schizophrenia and other psychoses by implementing evidence-based approaches in community settings. We present six lessons we have learned from this implementation using our application of the CBPR approach.

## Methods

### The setting

The network partners include the Adult and Older Adult Mental Health Services (AOAMHS) division of San Diego County and the Division of Geriatric Psychiatry of the University of California, San Diego (UCSD). AOAMHS provides public supported, linguistically and culturally appropriate mental health services for a large and diverse county equal in geographic and population size to the State of Connecticut (three million). Just over one-half of the clients (52.5%) are Caucasian, with 19.0% Latino, 11.3% African American, 4.8% Asian American, 0.6% Native American, and 11.8% mixed, other or unknown. Historically, no formalized structure was in place between UCSD and the county for the support of research, although some joint clinical projects have been conducted [9,10].

The partnership was developed from funds from the National Institute of Mental Health designated to support establishment of research networks. The initial goals of the partnership included needs assessment, utilization analysis, public education, and recruitment into research studies. Details of the outcomes of this partnership have been described elsewhere [11]. Briefly, however, the partnership has accomplished many significant outcomes. For example, the partners have conducted and disseminated a system-wide needs analysis of health services for older adults [12]; investigated the use of mental health services by gender, ethnicity, age, psychiatric diagnosis, and housing status [13-18]; provided several educational events, including a "miniconference" to the 2005 White House Conference on Aging. Moreover, to increase collaboration a community advisory board was formed to solicit stakeholder input at the study design phase of the research process, and the partners formed a coalition to provide education and advocacy for older adult mental health needs that has become a formal program of National Alliance on Mental Illness-San Diego. Here we highlight the processes by which the collaboration was developed and maintained, and the lesson we have learned.

### Developing the infrastructure for implementation

Public-academic partnerships combine two very different organizational systems, each with its own goals, values, styles, limitations, and pressures [19]. For example, the goal of community mental health systems is to efficiently provide effective treatments to those with psychiatric disorders with accountability to consumers, families, and taxpayers. In contrast, academic institutions conduct academically rigorous investigations of treatments with accountability to grant agencies, peers, and promotion committees. Therefore, not only do the types of data differ between these organizations, but also the method by which they collect, analyze, and interpret data vary. The balance between research and action diverges, as do timeframes and methods for demonstrating success. To address these organizational differences, we approached the formation of the partnership as an exercise in "cultural exchange" that occurs when different groups engage in a process of debate and compromise [20] to achieve a valued goal [21]. The process is necessarily bi-directional; both parties contribute, and both derive benefit. We report, below, on the lessons learned throughout the processes by which we became familiar with each other's organization (i.e., goals, values, styles, limitation, and pressures) that permitted us to accomplish mutually identified priorities.

## Results and discussion

### Lesson one. Changing attitudes

In developing this implementation network, concerns were raised with respect to liability, confidentiality, and added responsibilities for already busy clinical and research staff. As is often reported to be the case with university-community ties [22-24], academic researchers often found the additional bureaucratic processes that are necessary in public service organizations to be cumbersome. Then, too, county staff had difficulty with the university's organizational and administrative systems. Moreover, previous interactions had created a set of expectations and barriers that needed to be overcome to achieve a more effective partnership [8,19]. For example, sustainability of interventions in the community were not addressed, often leading to a loss of services on which the county had come to rely. Also, researchers were often not fully aware of the impact that the implementation of interventions had on county resources, nor did they appreciate the numerous levels of accountability for which the county was responsible (i.e., clients, providers, tax payers).

Forming a collaborative and productive partnership in the face of such barriers is complex and time-consuming and requires mutual trust and respect; changing these pre-existing attitudes were the initial focus of the relationship [6,23,25].

Prior to the formation of this partnership, AOAMHS and the university had no formal research collaborations, although the organizations had jointly participated in delivering some clinical services (e.g., psychiatric care for homeless mentally ill). Previous informal research relationships, however, had resulted in tension and doubt about the development of a truly collaborative research endeavor. It was essential to address these concerns, and change the attitudes of both partners at the outset.

As part of addressing organizational differences and formalizing the structure for the partnership, we held an initial series of four meetings in the first few months of the partnership, alternating between sites to educate each partner about the other's culture. These meetings consisted of presentations by leadership and staff from both institutions, and discussions on areas of overlap and mutual benefit. Using the process of consensus, it was decided that the partnership would focus on the following areas: needs analysis, education, service utilization review, and recruitment into specific study protocols.

We originally structured the partnership with three levels, each with parallel representation from each organization: staff, administrative, and executive teams. Originally, partnership staff met weekly or biweekly to discuss operations and projects. Higher level leadership from the county and the university joined staff monthly to address broader policy issues, resource allocation, and other administrative tasks. The county directors and the director of the research center joined the group quarterly for executive meetings to review progress and determine operational and research priorities. Over time, however, we found that the administrative and executive meetings were adequate for oversight and coordination, and discontinued the more frequent staff meetings replacing them with regular meetings to discuss scientific progress. These meetings were held monthly and included investigators, county staff, and the jointly hired personnel. Consistent with the cultural exchange model, not only did each organization change as the result of the transaction between the partners, but also the jointly established structures (i.e., staff meetings) were modified as the needs of the collaboration evolved.

### Lesson two. Sharing staff

Another lesson that required immediate attention was determining the allocation of resources specifically dedicated to the formation of the partnership. Both AOAMHS and the university recognized that personnel committed to the partnership development were an important investment. To increase communication, to assist in the understanding of each other's culture, and to foster cohesiveness, we jointly hired staff specifically for the partnership. The NIMH-funded center grant provided funds for a community mental health liaison and a data analyst to provide support to the county, who were housed at county offices for the express purpose of increasing communication. We also hired a research assistant whose time was shared between the UCSD and the county to aid in the development of reports and educational materials. Also, when the state budget crisis threatened the funding for the AMHS-funded position of the 'Older Adult Mental Health Coordinator', the partnership assumed financial responsibility for that position.

The jointly hired personnel, as well as staff from each organization, collaborated to ensure equal representation in all aspects of research, which is consistent with the principles CBPR. Education programs targeting the various stakeholder groups were developed and implemented. For example, an initiative identified by the county prior to the partnership was to provide a major educational program, 'The Wellness Campaign', for the general public. We collaboratively developed of a series of lectures given by national experts on such topics as the prevalence of psychiatric disorders, mental health assessment, depression and suicide, and psychopharmacological treatment in older adults. A broad audience of as many as 100 attendees, including researchers, providers, advocates, caregivers, and consumers, heard presentations at various accessible venues throughout the county, including senior centers and other community meeting sites. The program included a formal presentation and discussion, distribution of informational materials, and opportunities for networking.

In addition to increasing awareness of mental health issues of older persons to the general community, joint staff members made presentations to staff of various local and state agencies responsible for health and social services. Topics have included introducing evidence-based practices at a statewide meeting of county mental health directors and describing the nature and benefits of public-academic partnerships to researchers, and offering a 'refresher' course on research concepts for agency providers. In addition, senior university psychiatrists have been speakers for county-sponsored continuing medical education programs for physicians. Finally, the center, as well as AOAMHS, was co-sponsors of a consumer forum on late-life mental illness held in the spring of 2005 that was organized by the Geriatric Mental Health Foundation to gather input as part of a White House Conference on Aging.

Center staff members have also been instrumental in developing a new community-based, cooperative coalition, the Senior Wellness Coalition – San Diego. The coalition has been awarded a $34,000 California Endowment Foundation Grant to support its work in capacity-building and coordination. The center partnership also maintains representation on an Older Adult System of Care Council that provides recommendations to the local mental health director.

These educational activities, which indirectly pertain to research, required a substantial amount of the partnership resources and time. Inherent tensions associated with different emphases on tasks and processes are a common obstacle faced by partnerships [26], and the time needed to complete some tasks can be a major barrier to achieving partnership objectives [25]. Yet, these outreach efforts have resulted in numerous tangible benefits. The center and the county continued to gain knowledge about the other's culture through the implementation of these programs. Trust and respect were enhanced through an equitable distribution of decision-making and responsibilities.

### Lesson three. Expecting obstacles and formalizing solutions

As successful, independent operations, both the county and the center have developed strategies for solving problems and overcoming barriers. In the development of the partnership, however, fiscal and administrative problems emerged that neither organization anticipated. For example, we initially planned to have the county administer the budget for the partnership through a subcontract with the university. Because some university groups held contracts with the county to provide clinical services, AOAMHS could enter into a contract in which it received funds from the univerisity, even those funds provided by NIMH and designated specifically for the purposes of the collaboration. In order to progress with the development of the partnership, these unanticipated administrative and procedural issues had to be resolved. Through negotiation and compromise, requiring that each institution look beyond its distinct set of organizational priorities and loyalties [27,28], the partners decided that the university would manage the entire budget. The university became the designated employer of all staff, and the staff located at the county sites obtained 'volunteer' status. Both partners, however, retained joint determination of budget allocations, personnel selection, and supervision. This agreement and others were documented in a formal memorandum of understanding that outlined the terms of the collaboration and provided for annual review and revision, if necessary. The memorandum was developed with NIMH input and submitted as a formal amendment to the center grant award.

Another obstacle encountered by the partnership was recruitment of county participants into ongoing and new study protocols to increase the representation of our research samples. The shared staff facilitated identification of new recruitment sources and reduced the time spent on duplicative administrative aspects of obtaining approval to recruit at different county-affiliated sites. A major initiative involved collaboration with the County Public Conservator's Office, which is responsible for persons judged to be in need of the extra protection of guardianship, to develop policies that would allow participation of such individuals in minimal risk research projects. In the past, persons under public conservatorship were not permitted to participate in any type of research. University staff involved in the partnership, as well as the jointly hired staff, approached the director of the conservatorship program to explain the nature of the research projects, human subjects issues (i.e., the informed consent process, and minimum risk protocols), and the partnership itself. The director was invited to participate in executive staff meetings during which top county officials expressed their endorsement of partnership, and the collaborative and thorough nature of the process was demonstrated. The director agreed to modify the policy and allow the enrollment of conservatorized persons (with individual assent and conservator consent) in minimal-risk research as defined in 45CFR46 [29], helping to make the study samples more representative and increasing the potential applicability of findings to clinically fragile or disabled individuals. At that time, there were nine research protocols in which about 30 publicly conservatorized persons were participating.

The formation of a CBPR partnership by definition involves agencies with differing styles and procedures. The success of the collaboration depends heavily on the ability of the partners to anticipate and address obstacles in ways that each may have never previously considered.

### Lesson four. Monitoring and evaluating

UCSD and the county each had institutional mechanisms to track research projects. The formal mechanisms included the University's Human Research Protections programs and the County Research Committee. Because the county had limited capacity to review and monitor projects, the number of projects active in county programs was restricted. The partners worked to harmonize these processes in order to reduce the burden on the organizations and investigators, and created a database to track projects from initial proposal through study completion. Other databases were created to track subject participation and publications and reports. In general, the organizational expertise of the county complemented the scientific expertise of the academic investigators to create a monitoring and evaluation structure that assists investigators in the preparation of necessary documents, that reduces the demand on county resources, and that mitigates excessive subject burden by tracking the research participation of county clients.

The ability to monitor and evaluate outcomes and progress proved to be a crucial task of the partnership. The values of each organization differ, as do the methods employed to ensure the adherence to them; therefore, establishing mechanisms to monitor jointly agreed upon goals greatly facilitates communication and cohesion between the partners.

### Lesson five. Adapting and adjusting

Public-academic partnerships are established within a fluid context of political processes, changing priorities, and other events all of which require a flexible and adaptive approach not typically required in academic research. The partnership encountered three such challenges: changes of the county leadership, a natural disaster, and significant budget cutbacks. Each of these resulted in a resetting of project timetables that allowed staff to accommodate to the requirements of the moment. That the partnership survived and flourished indicates the strength of the arrangement and the validity of the pursuit. A good example is the implementation of the Privacy Rule of the Health Insurance Portability and Accountability Act (HIPAA) [30] regulating the use of medical data. This necessitated development of a new data use agreement to ensure that the data transfer between the partners was HIPAA-compliant. This agreement enabled investigators to retain access to de-identified information from the county's database, and a number of reports and publications have resulted.

Consistent with CBPR, the needs of both partners were equal. This necessitated at times that one partner had to re-evaluate and modify priorities in response to the other partner's issues. Moreover, both partners contributed equally to determining solutions to changes. These processes took much time and effort and may have slowed progress, but the result was a solution that satisfied both the county and the center.

### Lesson six. Taking advantage of emerging opportunities

In November 2004, Californians passed Proposition 63, the Mental Health Services Act (MHSA). The MHSA generates new tax revenue specifically earmarked to expand mental health services for the seriously mentally ill. The guiding purpose of this program was to transform the delivery of mental health services in California by instituting a recovery-oriented vision for new and expanded services and placing these services into the real world of homes, peer-run centers, clinics, and schools. For San Diego County, this has resulted in a budget increase of nearly $29 million through fiscal year 2007/2008. One of the key features of the MHSA was that each county was required to prioritize its own mental health needs, and in collaboration with a range of stakeholders, including consumers and family members, providers, and advocates, determine how the money would be used, emphasizing the need to deliver comprehensive services to a limited number of people rather than just broadly increasing services across the whole system. The UCSD-county partnership was instrumental in gathering, consolidating and analyzing stakeholder input and in conducting service utilization analyses that formed the core of the San Diego plan. which was approved with highest enthusiasm by the state's review committees.

The passage of the MHSA was not anticipated when the initial objectives of the partnership were selected. Nonetheless, the synergy of the partnership created several opportunities to further its goals and those of the MHSA. For example, we jointly conducted a needs assessment that not only fulfilled one of the goals of the partnership, but also provided important information for the planning of MHSA funds.

### Outcomes and benefits

Along with special analyses that were prepared as part of the county's MHSA application, investigators in the partnership have collaborated to complete nine studies; eight of these have been published on topics such as gender differences [13], ethnic disparities [14], and diagnostic- and age-related factors [15,16]. affecting service utilization for patients with schizophrenia, risk factors for homelessness [17], and the differential occurrence of substance and alcohol use disorders among different ethnic groups [31]. We conducted two studies linking the county's database with state Medicaid data. In one study, we found that residents of assisted care facilities had greater use of outpatient mental health services and lower rates of psychiatric and non-psychiatric hospitalization [18]. In the other study, we found that 41% of patients with schizophrenia were fully adherent and 16% were partially adherent to their prescribed antipsychotic drug schedule, and that both psychiatric and medical hospitalizations were strongly related to the degree of drug adherence [32]. The center provided a unique environment for the combination of academic and programmatic expertise necessary to pursue these analyses, which yielded valuable information for mental health services researchers and administrators.

## Conclusion

In establishing a network for implementation between an academic center and public mental health system based on CBPR, we encountered several issues that may generalize beyond our goal of the development and implementation of state-of-the-art approaches to assessment and interventions to improve the functioning and quality of life for persons with serious mental illnesses. The set of six "lessons learned" – changing attitudes; sharing staff; expecting obstacles and formalizing solutions; monitoring and evaluating; adapting and adjusting; and taking advantage of emerging opportunities – most likely will be applicable to the formation of other partnerships designed to provide necessary organizational support to facilitate the moving of the results of clinical research into community practice.

Starting with successful models of other academic-public collaborations [5,8]. and modifying them to the specific needs of the partners and the population, UCSD and San Diego County created a partnership focused on older adults with psychosis. The organizing rationale for this center was to establish an evidence-based partnership approach that adopted the principles of community-based participatory research in order to facilitate implementation of evidence-based approaches to assessment and intervention. The cultural exchange between two organizations that differed vastly in values orientations, bureaucracy, and function required a substantial investment of time, a strong commitment to the process, an openness to change, flexibility in the face of shifting contexts and priorities, and willingness to compromise and accommodate. The partnership received the endorsement of the top leadership in both organizations, an important factor in promoting cohesiveness and cooperation. Through this process, San Diego County has developed an infrastructure to support research, educational and advocacy programs (i.e., Senior Mental Health Coalition), and the furthered the development of the mental health delivery system for older adults. For example, MHSA funds support a mobile outreach team for older adults, a need that was identified through the needs assessment. The university has gained knowledge and awareness of community mental health services conditions, and improved its ability to develop and implement effective community-based participatory research projects for older persons with serious mental illnesses.

## Competing interests

The authors declare that they have no competing interests.

## Authors' contributions

LAL, BL, and RLH were responsible for the initial conceptualization and writing of the manuscript. PG, AA, MCH, CD, DVJ have been involved in revising the manuscript and adding substantial intellectual content. All authors read and approved the final manuscript.
